# Recovery capital: a concept analysis

**DOI:** 10.3389/fpubh.2026.1834864

**Published:** 2026-07-24

**Authors:** Yudie Zhang, Jun Wu, Wenting Xu, Fang Yang, Jianfen Ma

**Affiliations:** Affiliated Wuxi People's Hospital of Nanjing Medical University, Wuxi People's Hospital, Wuxi Medical Center, Nanjing Medical University, Wuxi, Jiangsu, China

**Keywords:** chronic disease management, concept analysis, healthy aging, public health, recovery capital

## Abstract

**Background:**

Recovery capital has emerged as an important concept for understanding how individuals sustain recovery and improve health outcomes across addiction recovery, chronic disease management, and healthy aging. However, the concept remains inconsistently defined, and its conceptual structure has not been fully clarified.

**Methods:**

This study used Walker and Avant's concept analysis method to synthesize the conceptual characteristics of recovery capital. Literature was systematically searched in PubMed and Web of Science from database inception to January 5, 2026. Following screening and eligibility assessment, 28 articles were included in the final analysis.

**Results:**

Recovery capital was conceptualized as a dynamic, strengths-based, multidimensional, and individualized construct involving the interaction of personal, relational, and contextual resources throughout recovery processes. The defining attributes of recovery capital included strengths-based resources, personalization, multidimensionality, interaction, and dynamic development. Antecedents included the presence of recovery-related threats, catalytic events initiating recovery processes, availability of supportive resources, and temporal conditions for resource accumulation. Consequences involved multidimensional restoration of functioning, psycho-social reconstruction and empowerment, improvement in long-term health outcomes and quality of life, and the discovery of meaning and purpose in life.

**Conclusion:**

This concept analysis provided a synthesized understanding of recovery capital and clarified its defining attributes, antecedents, consequences, empirical referents, and conceptual boundaries. The findings may contribute to the conceptual development and interdisciplinary application of recovery capital in healthy aging, with particular relevance for maintaining functional ability and social participation in aging populations. Further research is needed to operationalize and validate recovery capital in the older adults.

## Introduction

The term “recovery” is widely used in the research literature and in clinical settings. As early as 1982, the American Society of Addiction Medicine (ASAM) defined recovery as reaching “a state of physical and psychological health such that abstinence from dependency-producing drugs is complete and comfortable” (1). In 2007, Substance Abuse and Mental Health Services Administration defined recovery from alcohol and drug problems as “a process of change through which an individual achieves abstinence and improved health, wellness and quality of life” (2, 3). In mental health research, Tew J proposed that “life recovery” means regaining a sense of control over one's life and finding a satisfying and valuable life position for oneself (4). Dimitrios and colleagues conducted a study to explore the definition of ‘recovery' for delirium and proposed that in the older adluts, a full recovery may never be achieved, it is perhaps better to define recovery according to a symptomatic status that can be measured by a variety of diagnostic instruments (5). In 2021, Cieza and colleagues characterized recovery as a complex, multidimensional, and dynamic process across the entire lifespan and population groups, with its core focus on restoring health, function, and dignity (6). A mixed-method systematic review conducted by Lukacs and colleagues in 2024 explored how recovery was defined and measured among patients with low back pain. The review found that more than one-third of published studies lacked a formal definition of recovery (7). Consequently, the meaning of recovery varies substantially across disciplines and clinical contexts.

Recovery capital is fundamentally grounded in the concept of recovery; therefore, changes in how recovery is understood may directly influence the interpretation and application of recovery capital. As the concept of recovery has expanded across addiction medicine, mental health, healthy aging, and chronic disease management, the conceptualization of recovery capital has also evolved, resulting in increasing conceptual ambiguity and inconsistency in its definition and application. The concept of recovery capital was first proposed by Granfield and Cloud in 1999 within the context of addiction recovery. Grounded in the biopsychosocial model, it was used to describe the personal and social resources individuals may draw upon to overcome substance misuse (8). Over two decades have passed since the term's first introduction. However, the conceptualization of recovery capital remains inconsistent across the literature, particularly regarding whether it should be understood as exclusively strengths-based or whether it may also encompass negative dimensions. Such conceptual ambiguity may hinder the theoretical development, empirical measurement, and practical application of recovery capital (9).

This issue is particularly relevant in the context of healthy aging and chronic disease management. Healthy aging is defined as the process of developing and maintaining the functional ability that enables wellbeing in older age (10). Increased longevity provides older adults with opportunities to reconsider how later life may unfold (10). However, aging is also associated with an increased risk of multimorbidity (11). In current clinical reasoning, when facing diverse physical and psychological responses among older adults under frequent and multiple health stressors, what needs to be addressed is not only their diseases, but also whether they are able to recover from such “adversities” (12). The recovery process in later life is deeply embedded in the resources available to individuals. Whether older adults have access to and are able to mobilize internal and external resources is of critical importance for their recovery outcomes (13). Older adults often experience age-related functional decline, frailty, and reductions in intrinsic capacity, which further complicate recovery processes and highlight the need for resource-based perspectives (14, 15). Recovery capital may therefore serve as a key resource framework for understanding how older adults mobilize personal and external resources across the aging life-course to maintain functional ability and social participation. Accordingly, examining recovery capital in older populations may provide important insights for public health and geriatric nursing practice.

Given the conceptual inconsistency surrounding recovery capital, a clearer conceptual understanding is needed to support theoretical development, empirical measurement, and interdisciplinary application. This need is particularly urgent in the context of population aging and the increasing prevalence of chronic and multimorbid conditions, where recovery processes are closely linked to functional decline, frailty, and long-term care needs (13). From a public health and gerontological perspective, clarifying how recovery capital is defined and operationalized may help better understand how older adults mobilize resources to maintain functional ability and social participation across the aging life-course. Concept analysis provides a systematic approach for clarifying ambiguous concepts and identifying their defining attributes, antecedents, and consequences (16). Therefore, this study aimed to clarify the concept of recovery capital using Walker and Avant's concept analysis method, explicitly focusing on its application within aging populations and its implications for public health and gerontological practice.

## Methods

### Design

This concept analysis was conducted based on Walker and Avant's method (17), consisting of the following steps: (1) Selecting the concept; (2) Determining the aims of the analysis; (3) Identifying all possible uses of the concept; (4) Determining the defining attributes of the concept; (5) Identifying a model case; (6) Identifying borderline, related, and contrary cases; (7) Identifying antecedents and consequences; (8) Identifying empirical referents of the concept.

### Data sources

Literature relevant to the concept of recovery capital was searched in PubMed and Web of Science databases covering all publications up to January 5, 2026. The following search terms were used in various combinations: “recovery capital,” “rehabilitation capital,” “recovery resource,” and “rehabilitation resource”. The term “rehabilitation” was included to capture conceptually related literature potentially relevant to recovery capital.

The inclusion criteria were: (1) articles written in English, (2) original research studies, systematic reviews, concept analyses, and theoretical or qualitative studies that explicitly discussed the definition, characteristics, development, application, or conceptualization of recovery capital. The exclusion criteria were: (1) incomplete or inaccessible full-text articles; (2) conference papers, editorials, and letters to the editor. Additionally, reference lists of selected articles were hand-searched to identify further relevant literature. Two nursing researchers independently screened the literature, and any discrepancies were resolved through discussion with a third researcher.

**Figure 1:**
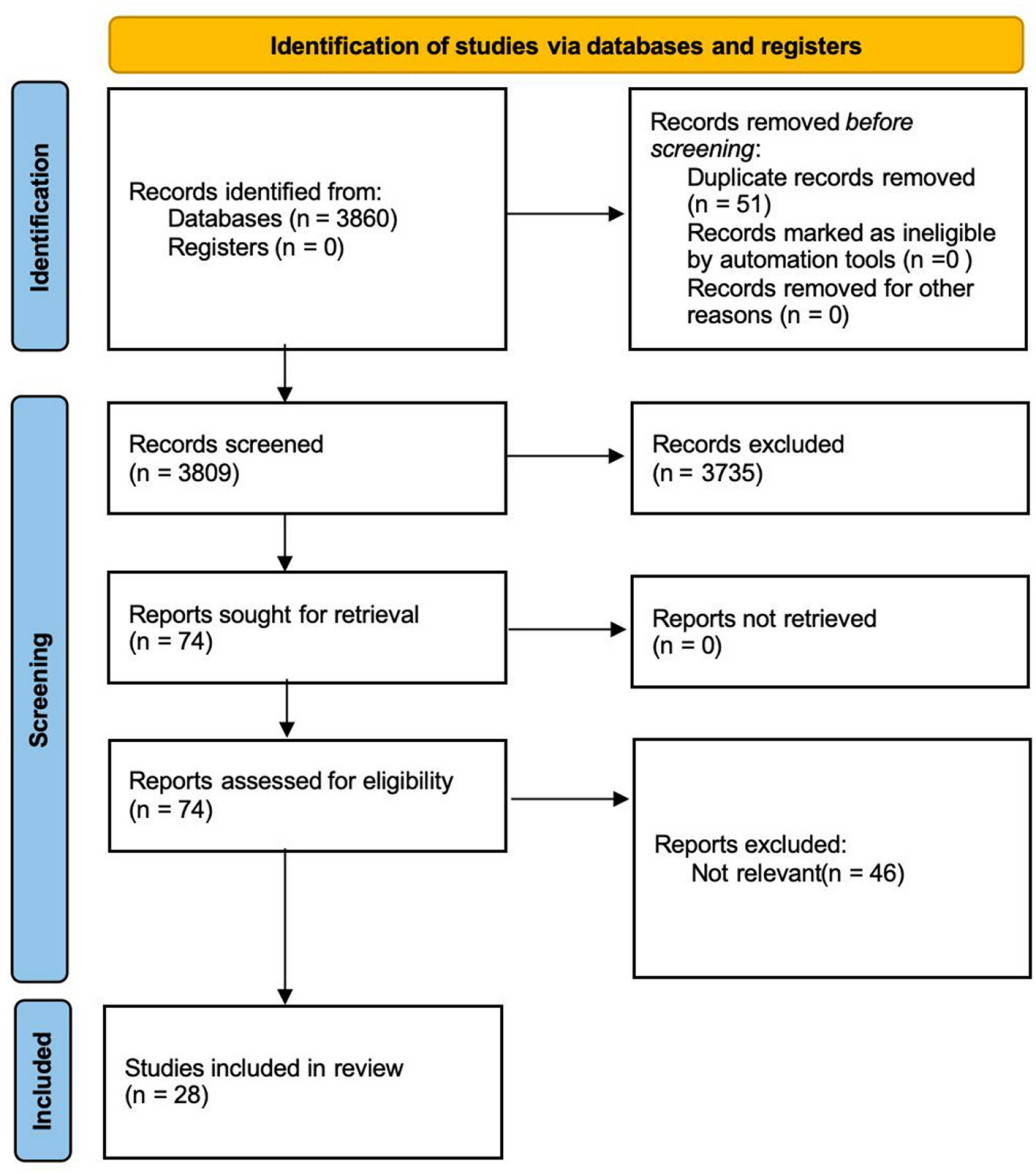
PRISMA flow diagram illustrating the article selection process.

The initial database search included 3,860 records. After removing duplicates, 3,809 articles remained for preliminary screening. Following the screening of titles and abstracts, 74 full-text articles were assessed for eligibility. A further 46 articles were excluded after full-text review for various reasons. Finally, 28 articles were included in the final analysis and were subjected to a thorough review using Walker and Avant's concept analysis framework. The literature screening process is illustrated in Figure 1, and detailed information on the included literature is provided in Supplementary Table S1.

### Data analysis

The included literature was analyzed following Walker and Avant's concept analysis framework. Data related to the definitions, characteristics, antecedents, consequences, and empirical referents of recovery capital were extracted from each study. Recurring conceptual characteristics across the included studies were identified through iterative reading and comparison. Similar concepts and descriptions were grouped into preliminary categories, which were subsequently refined into defining attributes through discussion and consensus among the research team. Antecedents and consequences were identified by examining conditions that preceded the development of recovery capital and outcomes associated with its presence across the literature. Any discrepancies in interpretation were resolved through discussion until consensus was reached.

## Results

### Identifying all possible uses of the concept

“Recovery capital” is a phrasal noun comprising two sub-concepts: “recovery” and “capital”. Dictionary definitions generally describe “recovery” as a process of regaining health or overcoming difficulties, while “capital” refers to useful assets, resources, or advantages (18, 19).

For older adults undergoing recovery, recovery capital serves as an important foundation for achieving improvements in health, function, dignity, and social reintegration (9). From the perspective of health and nursing professionals, assessing recovery capital that support the maintenance of functional independence and social participation in later life, thereby improving recovery trajectories among older adults (20). Existing studies emphasized the holistic influence of multiple resources on recovery and highlighted the importance of personal, relational, and contextual support systems in promoting long-term recovery outcomes, particularly in populations with complex health needs in later life (21).

### Defining attributes of recovery capital

#### Strengths-based Resources

Across the included literature, recovery capital was commonly conceptualized as a strengths-based construct encompassing resources that support individuals throughout the recovery process (22). The majority of studies described recovery capital as involving positive internal and external resources that facilitate recovery, adaptation, and social reintegration (9, 20, 21, 23–26). Although a minority of studies discussed the possibility of “negative recovery capital,” referring to resources or conditions that may obstruct recovery processes (27–29), strengths-based resources remained the most consistently emphasized characteristic across the included literature.

#### Personalized

Across the included studies, recovery capital was frequently described as personalized and individualized (23). Individuals differed in their access to resources, recovery goals, social environments, and health conditions, resulting in varying recovery needs and pathways (24). Therefore, the development and mobilization of recovery capital may vary according to personal circumstances and stages of recovery.

#### Individual, relational, and contextual

Across the included literature, recovery capital was consistently conceptualized as a multidimensional construct comprising individual, relational, and contextual resources. Individual resources commonly included education, physical health, financial assets, and psychological strengths (24–26, 30–32). Relational resources involved supportive interpersonal relationships and social interactions, such as intergenerational support, peer mutual assistance, and marital relationships (4, 20, 24, 30, 33, 34). Contextual resources included broader environmental and structural supports, such as community environments, policy support, and healthcare security systems (9, 23, 24, 26–29, 33, 34).

#### Interactive

The included studies suggested that different components of recovery capital interact and influence one another (9, 30). The combined effects of personal, relational, and contextual resources may either facilitate or hinder individuals' recovery processes, reflecting the interconnected nature of recovery capital across multiple domains.

#### Dynamic

Recovery capital was frequently described as dynamic and changeable across time, settings, and stages of recovery (32). Several studies emphasized that recovery capital may increase, decrease, or fluctuate according to individuals' life experiences, health conditions, and social environments (9, 20, 24, 35, 36). This dynamic nature highlights the importance of continuously assessing recovery-related resources throughout the recovery process.

### Model case

An older adult experiencing functional decline and reduced social participation following multiple chronic conditions recognized the need to restore health and maintain independence in daily living. The individual possessed personal resources, including health literacy, adaptive coping strategies, and self-management capacity (demonstrating strengths-based and personalized resources). Relational resources were reflected through emotional and instrumental support from family caregivers and participation in community-based older adults' support networks, while contextual resources included access to primary healthcare services, community rehabilitation programs, and age-friendly social support systems (demonstrating multidimensional recovery resources). These resources interacted dynamically throughout the recovery process. Family support facilitated adherence to rehabilitation and health management plans, while community services enhanced opportunities for social participation and functional maintenance (demonstrating interaction among personal, relational, and contextual resources). Over time, the individual reported improved functional ability, enhanced self-care independence, strengthened social engagement, and improved overall wellbeing (reflecting the dynamic development of recovery capital in later life). This case demonstrates the defining attributes of recovery capital, including strengths-based resources, personalization, multidimensionality, interaction, and dynamic development, within the context of healthy aging.

### Borderline, related, and contrary cases

#### Borderline case

An older man experiencing relocation-related loneliness possessed certain individual and contextual resources, including stable financial support, preserved cognitive function, and access to community facilities. He was able to maintain basic daily functioning and adapt to environmental changes (partially demonstrating strengths-based and contextual resources). However, he showed limited initiative in social participation and maintained minimal contact with family members. Interactions among available resources remained weak, and supportive resources were not effectively mobilized to promote recovery (limited interaction and dynamic development). Although some recovery-related resources were present, the older man failed to achieve meaningful improvements in psychological wellbeing or social reintegration, reflecting only partial manifestation of recovery capital.

#### Related case

An individual with chronic disease participated in a structured hospital-led disease management program and regularly received medical follow-up, medication adjustment, and health education. Formal healthcare support was available, and the individual passively adhered to medical recommendations (demonstrating partial contextual support). However, recovery-related resources remained primarily limited to clinical management. The individual lacked active resource mobilization, social participation, peer interaction, and adaptive recovery strategies (lacking multidimensional interaction and personalization). This case reflects disease management and treatment adherence rather than recovery capital.

#### Contrary case

A long-retired older man reported persistent hopelessness, social withdrawal, and loss of life purpose. The man lacked supportive interpersonal relationships, financial resources, community engagement, and professional support (absence of individual, relational, and contextual resources). No active attempts were made to seek help or mobilize available resources, and psychological distress progressively worsened over time (absence of dynamic development and interaction among resources). This case lacks the defining attributes of recovery capital.

### Antecedents and consequences of recovery capital

#### Antecedents

Antecedents of recovery capital refer to conditions or circumstances that facilitate the emergence and development of recovery capital.

##### Presence of threats

Across the included literature, recovery capital commonly emerged in the context of physiological, psychological, or social challenges requiring recovery-related adaptation (20, 23). These threats included illness, emotional distress, social impairment, dissatisfaction with life or health status, and other adverse conditions that created the need for recovery.

##### Catalytic events initiating the recovery process

Specific situational or experiential events often serve as turning points that prompt individuals to enter a recovery pathway. These catalysts may include receiving a formal diagnosis, discharge from acute care, significant life transitions, or encounters that heighten awareness of health risks (20, 24, 26).

##### Availability of supportive resources

For recovery capital to emerge or be mobilized, individuals must have access to supportive resources situated within both internal and external environments. The availability of such resources was identified as an important precondition for the development of recovery capital (9, 27).

##### Temporal conditions for resource accumulation

The formation and growth of recovery capital are not instantaneous. The accumulation and mobilization of recovery-related resources require time for individuals to recognize, access, integrate, and apply supportive resources throughout the recovery process. In addition, the influence of catalytic events and supportive environments may progressively contribute to the development of recovery capital over time (24, 36).

#### Consequences

##### Multidimensional restoration of functional capacity

Recovery capital was frequently associated with the improvement of physical, psychological, and social functioning during the recovery process, as well as the re-establishment of family and social roles (20, 22, 37).

##### Psycho-social reconstruction and empowerment

Beyond functional improvement, recovery capital may contribute to a renewed sense of personal agency and control over life circumstances. This process is accompanied by enhanced hopefulness, dignity and optimism (4, 32, 36).

##### Improvement in long-term health outcomes and quality of life

As a strengths-based resource, recovery capital contributes, from a long-term perspective, to increasing health stability and enhancing individuals' quality of life, life satisfaction, and subjective wellbeing (4, 22, 37).

##### Discovery of meaning and purpose in life

Recovery capital may help individuals identify their purpose and meaning in life. By clarifying personal values and pursuing meaningful pursuits, individuals can establish a clear direction for their lives (4, 22, 36).

#### Empirical referents of recovery capital

Empirical referents of recovery capital include observable dimensions, measurable indicators, and conceptual frameworks that support the assessment and operationalization of recovery capital in practice. Across the included literature, recovery capital was commonly conceptualized as a multidimensional construct encompassing personal, social, cultural, physical, and contextual resources (4, 27, 37). Several theoretical frameworks have been developed to operationalize recovery capital in different recovery contexts. For instance, the theoretical model of recovery capital proposed by Cloud and colleagues conceptualizes recovery capital as comprising four structural dimensions: social capital, physical capital, human capital, and cultural capital (27). Kline and collaborators developed a recovery capital framework for individuals with eating disorders, suggesting that recovery capital encompasses social capital, physical capital, cultural capital, and personal and psychological health capital (37). In mental health recovery research, Tew and colleagues proposed a recovery capital framework to support mental health recovery, in which recovery capital includes economic capital, social capital, relational capital, identity capital, and personal capital (4).

Although the specific dimensions varied across frameworks (4, 27, 37), these models consistently emphasized the multidimensional and resource-oriented nature of recovery capital. In the context of population aging, these dimensions may be particularly relevant for assessing older adults' functional ability, social participation, and access to care resources, especially in the presence of multimorbidity and age-related functional decline. These refined structures and indicators offer useful references for understanding and operationalizing the concept of recovery capital and may support its measurement and validation in practice.

### Conceptualization of recovery capital

Recovery capital was conceptualized as a dynamic, strengths-oriented combination of internal and external assets that individuals are able to access, mobilize, and integrate during restorative processes. These resources include personal capacities, relational supports, and contextual conditions that interact across time, settings, and stages of recovery. Through their coordinated utilization, recovery capital may support older individuals in adapting to health challenges, sustaining recovery processes, restoring physical, psychological, and social functioning, and developing meaning and purpose in life (Table 1).

**Table 1:** Attribute, antecedents, and consequences of the concept.

Antecedents	Attributes	Consequences
Presence of threats	Strengths-based Resources	Multidimensional restoration of functional capacity
Catalytic events initiating the recovery process	Personalized	Psycho-social reconstruction and empowerment
Availability of supportive resources	Individual, Relational, and Contextual	Improvement in long-term health outcomes and quality of life
Temporal conditions for resource accumulation	Interactive	Discovery of meaning and purpose in life
	Dynamic	

## Discussion

Recovery capital has traditionally been discussed within addiction recovery contexts and was originally conceptualized as a strengths-oriented resource framework supporting individuals throughout recovery processes (27). However, the included literature revealed continuing conceptual variation regarding the nature and boundaries of recovery capital. While a minority of studies discussed the possibility of “negative recovery capital” (27–29, 38), the present analysis found that strengths-based resources remained the most consistently emphasized characteristic across the included literature (9, 20, 21, 23–26). Cloud proposed that recovery capital might, under certain circumstances, fall below zero and become “negative recovery capital,” referring to conditions that obstruct recovery processes (27). Nevertheless, the included literature predominantly conceptualized recovery capital as a strengths-oriented construct involving resources that facilitate adaptation, recovery, and social reintegration (23–26). This finding may reflect the tendency of existing literature to conceptualize recovery capital primarily as a resource-oriented and strengths-based construct.

The present analysis also highlighted the interactive and dynamic nature of recovery capital. Although relatively few previous studies explicitly discussed interactions among recovery-related resources, the included literature suggested that personal, relational, and contextual resources may influence one another throughout recovery processes (9, 30). The availability or imbalance of these resources may facilitate or hinder recovery depending on individuals' circumstances and resource configurations. In addition, recovery capital appeared to fluctuate across different recovery stages, settings, and life experiences (20, 23). As individuals' recovery needs evolve over time, the mobilization and significance of recovery-related resources may also change, reflecting the individualized nature of recovery capital. These findings suggest that recovery capital should not be understood as a static collection of resources, but rather as a dynamic process shaped by ongoing interactions between individuals and their environments (20, 23). In later life, these dynamic interactions are further shaped by age-related changes in intrinsic capacity, social network contraction, and increasing care dependency (12).

Previous studies have proposed multiple structural frameworks for recovery capital, reflecting the ongoing development and contextual diversity of the concept. Early models primarily emphasized personal, social, financial, and cultural dimensions (27), whereas subsequent studies further incorporated community, relational, emotional, and mental health-related resources (4, 9, 20, 37, 39). These variations suggest that the dimensional structure of recovery capital may differ across populations, recovery contexts, and theoretical perspectives. For example, mental health-related resources may receive greater emphasis in populations experiencing psychological distress, whereas community and financial resources may be more prominent in chronic illness or social reintegration contexts. The present analysis synthesized recovery capital as comprising personal, relational, and contextual resources that interact throughout recovery processes, providing a conceptual perspective applicable in recovery-related aging fields.

In the context of healthy aging, recovery capital may play an important role in supporting the maintenance of functional ability and psychological health. Beyond the challenges posed by multimorbidity and age-related physiological changes, older adults also experience unique psychological and social vulnerabilities, which may further increase their susceptibility to adverse health outcomes (40). To cope with these challenges, they mobilize various personal and social resources to obtain emotional and instrumental support. However, the mechanisms through which older adults dynamically mobilize and integrate resources to support recovery remain insufficiently understood. By emphasizing the interaction of personal, relational, and contextual resources, recovery capital may therefore provide a useful framework for understanding resource-based pathways to healthy aging. It may also guide recovery-oriented assessment and intervention development (35). In addition, recognizing the dynamic and individualized nature of recovery capital may support more flexible and person-centered recovery strategies across different recovery stages and settings.

The development of measurement instruments remains an important direction for future research. Existing recovery capital assessment tools have primarily been developed for populations with substance use disorders, and no universally validated instrument for older adult populations is currently available (38). Future studies should therefore further operationalize and validate recovery capital in older adults and aging populations. Longitudinal research may also help clarify the developmental trajectories of recovery capital and identify recovery-oriented strategies that support individuals in mobilizing strengths and resources throughout recovery processes.

### Limitations

This study has the following limitations. First, only literature retrieved from selected databases was included, which may have resulted in the omission of potentially relevant studies. Second, all the included articles were in English, which may have introduced language bias. Third, although this study synthesized the conceptual characteristics of recovery capital through Walker and Avant's concept analysis method, the identification and interpretation of defining attributes, antecedents, and consequences inevitably involved a degree of interpretive judgment. In addition, the conceptualization proposed in this study has not yet been empirically validated across diverse clinical and recovery contexts. Future research is needed to further operationalize and validate recovery capital among different populations and recovery settings.

## Conclusion

This concept analysis provided a synthesized understanding of the conceptual characteristics of recovery capital using Walker and Avant's method. Across the included literature, recovery capital was conceptualized as a dynamic, strengths-based, multidimensional, and individualized construct involving the interaction of personal, relational, and contextual resources throughout recovery processes. The analysis identified the defining attributes, antecedents, consequences, empirical referents, and conceptual boundaries of recovery capital, which may contribute to a clearer understanding and broader application of the concept in healthy aging.

Future research should further operationalize the definition of recovery capital for specific populations, particularly older adults and those experiencing age-related functional decline, and identify the key recovery resources for these groups. Additionally, researchers should explore the change trajectory of recovery capital, provide targeted intervention measures, integrate recovery capital into older adults' recovery journeys, and help individuals fully utilize their strengths and resources to facilitate their recovery processes.
