# Supplementary material for: Recovery capital: a concept analysis
Source: Front Public Health. 2026 Jul 24;14:1834864. doi: 10.3389/fpubh.2026.1834864 (PMC13447157; doi:10.3389/fpubh.2026.1834864)
Supplement: Supplementary file 1 [file Table_1.docx]

**Supplementary Table S1. A detailed overview of the included studies**

| Study ID | Author | Year | Title | Study Design | Aim | Integration Section |
| --- | --- | --- | --- | --- | --- | --- |
| 1 | Elswick A, et al. | 2018 | Emerging Adults and Recovery Capital: Barriers and Facilitators to Recovery | Qualitative research | To explore the composition of recovery capital among individuals aged 18–25 years with substance addiction | Determining the defining attributes |
| 2 | Moghanibashi-Mansourieh A, et al. | 2020 | The role of reflective consequences in developing recovery capital for the recovering substance abuser population of Tehran city | Qualitative research | To propose a theoretical framework of recovery capital applicable to people with substance addiction | Determining the defining attributes and consequences |
| 3 | Sterling R, et al. | 2008 | Measuring recovery capital and determining its relationship to outcome in an alcohol dependent sample | Tool development | To screen items from established scales for measuring recovery capital and examine its relationships with short-term and long-term outcomes | Determining the defining consequences |
| 4 | Best D, et al. | 2022 | The science of recovery capital: where do we go from here? | Narrative review | To clarify the concept of recovery capital among individuals with substance addiction | Determining the defining attributes |
| 5 | Best D, et al. | 2020 | Measuring capital in active addiction and recovery: the development of the strengths and barriers recovery scale (SABRS) | Tool development | To develop a tool for assessing the recovery strengths and barriers among individuals with substance addiction | Determining the defining attributes and antecedents |
| 6 | Bowen E, et al. | 2022 | Qualitative insights in item development for a comprehensive and inclusive measure of recovery capital | Qualitative research | To develop an item inventory of recovery capital for individuals with substance addiction | Determining the defining attributes |
| 7 | Bowen E, et al. | 2023 | Development and psychometric properties of the Multidimensional Inventory of Recovery Capital (MIRC) | Tool development | To develop a recovery capital measurement tool for individuals with alcohol addiction | Empirical referents |
| 8 | Bunaciu A, et al. | 2024 | Measuring recovery capital for people recovering from alcohol and drug addiction: A systematic review | Systematic review | To confirm the concept and measurement tools of recovery capital | Determining the defining attributes |
| 9 | Chen G. | 2018 | Building Recovery Capital: The Role of "Hitting Bottom" in Desistance and Recovery from Substance Abuse and Crime | Theoretical research | To explore the triggers for individuals to build recovery capital | Determining the defining attributes and antecedents |
| 10 | Cloud W, et al. | 2008 | Conceptualizing recovery capital: expansion of a theoretical construct | Theoretical research | To explore the concept of recovery capital among individuals with substance addiction | Determining the defining attributes |
| 11 | Francis MW, et al. | 2022 | Deriving a Measure of Social Recovery Capital From the Important People and Activities Instrument: Construction and Psychometric Properties | Tool development | To develop a measurement tool for social recovery capital | Determining the defining attributes |
| 12 | Gavriel-Fried B, et al. | 2021 | Negative Recovery Capital in Gambling Disorder: A Conceptual Model of Barriers to Recovery | Theoretical research | To explore the concept of recovery capital in Gambling Disorder | Determining the defining attributes |
| 13 | Gavriel-Fried B, et al. | 2022 | The Holistic Recovery Capital in Gambling Disorder index: A pilot study | Tool development | To develop The holistic recovery capital instrument in gambling disorder | Determining the defining attributes and consequences |
| 14 | Gavriel-Fried B, et al. | 2022 | Recovery capital in action: How is gender understood and employed by men and women recovering from gambling disorder? | Mixed methods research | To explore gender as a key factor of recovery capital | Determining the defining attributes |
| 15 | Groshkova T, et al. | 2012 | The Assessment of Recovery Capital: properties and psychometrics of a measure of addiction recovery strengths | Tool development | To develop a measurement tool for recovery capital among individuals with substance addiction | Determining the defining attributes |
| 16 | Hennessy EA, et al. | 2022 | Parenting to provide social recovery capital: A qualitative study | Qualitative research | To explore the social recovery capital of parents of adolescents with substance use disorders | Determining the defining attributes |
| 17 | Hindhede AL. | 2023 | Struggling with capital: Recovery after severe traumatic brain injury among working-age individuals in Denmark | Qualitative research | To explore the recovery capital of patients with traumatic brain injury (TBI) | Determining the defining attributes |
| 18 | Ingram C, et al. | 2025 | Barriers and enablers of addiction recovery amongst people experiencing homelessness in Dublin, Ireland: A proposed conceptual framework adapted from the REC-CAP | Qualitative research | To explore the recovery capital of homeless individuals with substance addiction. | Determining the defining attributes |
| 19 | Jadovich E, et al. | 2024 | Recovery is about change, so you have to change everything": Exploring the evolution of recovery capital among women in substance use disorder treatment | Qualitative research | To exploring the evolution of recovery capital among women in substance use disorder treatment | Determining the defining attributes |
| 20 | Pars E, et al. | 2023 | Using the Recovery Capital Model to Explore Barriers to and Facilitators of Recovery in Individuals with Substance Use Disorder, Psychiatric Comorbidity and Mild-to-Borderline Intellectual Disability: A Case Seriest | Case study | To explore barriers to and facilitators of recovery in individuals with substance use disorder, psychiatric comorbidity and mild-to-borderline intellectual disability | Determining the defining attributes |
| 21 | Pouille A, et al. | 2021 | Recovery Capital among Migrants and Ethnic Minorities in Recovery from Problem Substance Use: An Analysis of Lived Experiences | Qualitative research | To explore the recovery capital among migrants and ethnic minorities with substance use disorder | Determining the defining attributes |
| 22 | Pouille A, et al. | 2022 | Recovery capital among migrants and ethnic minorities: A qualitative systematic review of first-person perspectives | Systematic review | To examine the recovery capital among immigrant and minority populations. | Determining the defining attributes |
| 23 | Vilsaint CL, et al. | 2017 | Development and validation of a Brief Assessment of Recovery Capital (BARC-10) for alcohol and drug use disorder | Tool development | To develop a measurement instrument of recovery capital for people with alcohol and substance use disorders | Determining the defining attributes |
| 24 | Whitesock D, et al. | 2018 | Validating a Survey for Addiction Wellness: The Recovery Capital Index | Tool development | To develop a recovery capital index for people with alcohol and substance use disorders | Determining the defining attributes |
| 25 | Tew J. | 2012 | Recovery capital: what enables a sustainable recovery from mental health difficulties | Theoretical research | To explore recovery capital among individuals with mental health disorders. | Determining the defining attributes |
| 26 | Benight CC, et al. | 2018 | Self-Regulation Shift Theory: A Dynamic Personal Agency Approach to Recovery Capital and Methodological Suggestions | Theoretical research | To identify recovery capital among individuals with post-traumatic adaptation. | Determining the defining attributes |
| 27 | Kline KM. | 2023 | A conceptual framework for recovery from eating disorders: an adoptive approach to the recovery capital model | Theoretical research | To develop a theoretical framework of recovery capital among people with eating disorders | Determining the defining attributes |
| 28 | Nisic M, et al. | 2025 | What constitutes a women’s recovery from addiction? Examining lived experiences and recovery capital of women from diverse European backgrounds | Qualitative research | To explore the recovery capital of female patients with substance addiction | Determining the defining attributes |
